# The circadian syndrome is a better predictor for psoriasis than the metabolic syndrome via an explainable machine learning method — the NHANES survey during 2005–2006 and 2009–2014

**DOI:** 10.3389/fendo.2024.1379130

**Published:** 2024-06-26

**Authors:** Yunfan Gu, Xinglan Ye, Wenting Zhao, Shiwei He, Weiming Zhang, Xianyu Zeng

**Affiliations:** ^1^ Department of Dermatology, Traditional Chinese and Western Medicine Hospital of Wuhan, Tongji Medical college, Huazhong University of Science and Technology, Wuhan, Hubei, China; ^2^ Department of Dermatology, Suzhou Hospital of Traditional Chinese Medicine Affiliated to Nanjing University of Chinese Medicine, Suzhou, China; ^3^ School of Clinical Traditional Chinese Medicine, Hubei University of Chinese Medicine, Wuhan, China; ^4^ First Clinical College, Hubei University of Chinese Medicine, Wuhan, China

**Keywords:** metabolic syndrome, circadian syndrome, psoriasis, NHANES, machine learning, Mendelian randomization

## Abstract

**Objective:**

To explore the association between circadian syndrome (CircS) and Metabolic Syndrome (MetS) with psoriasis. Compare the performance of MetS and CircS in predicting psoriasis.

**Methods:**

An observational study used data from the NHANES surveys conducted in 2005–2006 and 2009–2014. We constructed three multiple logistic regression models to investigate the relationship between MetS, CircS, and their components with psoriasis. The performance of MetS and CircS in predicting psoriasis was compared using five machine-learning algorithms, and the best-performing model was explained via SHAP. Then, bidirectional Mendelian randomization analyses with the inverse variance weighted (IVW) as the primary method were employed to determine the causal effects of each component.

**Result:**

A total of 9,531 participants were eligible for the study. Both the MetS (OR = 1.53, 95%CI: 1.07–2.17, *P* = 0.02) and CircS (OR = 1.40, 95%CI: 1.02–1.91, *P* = 0.039) positively correlated with psoriasis. Each CircS algorithmic model performs better than MetS, with Categorical Features+Gradient Boosting for CircS (the area under the precision-recall curve = 0.969) having the best prediction effect on psoriasis. Among the components of CircS, elevated blood pressure, depression symptoms, elevated waist circumference (WC), and short sleep contributed more to predicting psoriasis. Under the IVW methods, there were significant causal relationships between WC (OR = 1.52, 95%CI: 1.34−1.73, P = 1.35e-10), hypertension (OR = 1.68, 95%CI: 1.19−2.37, P = 0.003), depression symptoms (OR = 1.39, 95%CI: 1.17−1.65, P = 1.51e-4), and short sleep (OR = 2.03, 95%CI: 1.21–3.39, p = 0.007) with psoriasis risk.

**Conclusion:**

CircS demonstrated superior predictive ability for prevalent psoriasis compared to MetS, with elevated blood pressure, depression symptoms, and elevated WC contributing more to the prediction.

## 1 Introduction

Psoriasis is an autoimmune disorder that affects 2% of the world’s population (1). It is caused by the interaction of several factors, including immune system disorders, inflammatory mediators from different pathways, autoantigens, psoriasis-associated susceptibility genes, and stimuli from various environmental factors (2), leading to the overactivation of immune cells and chronic inflammation. With the increasing pressure of life in modern society, the incidence and prevalence of psoriasis are increasing yearly (3). The intrinsic risk factors of psoriasis, such as hypertension, obesity, diabetes mellitus, endocrine disorder, and stress, received more attention (4, 5). Metabolic syndrome (MetS), which includes these risk factors, can be a suitable generalization of metabolic disorders in the human body. Insulin resistance, oxidative stress, and central obesity-related inflammation caused by MetS are all inextricably interconnected with the development of psoriasis (6).

Lately, researchers have found that circadian rhythm disruptions, including sleep disorders and depression, exacerbate psoriasis (7, 8) and are often associated with metabolic syndrome (9). Nonetheless, these risk factors are often viewed as separate components and not taken into account in connection with MetS. The unhealthy lifestyle of modern society (including a sedentary lifestyle, lack of exercise, constant anxiety and depression, lack of sleep, exposure to light and noise, and high-calorie food) has led to not only metabolic disorders but also circadian imbalances (10). MetS alone hardly explains these metabolic imbalances caused by circadian rhythm disruption. A new concept, the circadian syndrome (CircS), has been proposed to harmonize these factors (11). CircS combines depression symptoms and short sleep based on the components of MetS. It has been found to be strongly related to coronary heart disease, stroke, and endocrine disruption (12–14). Therefore, we hypothesize that CircS should be considered a new psoriasis risk group besides MetS and may be able to represent psoriasis caused by unhealthy lifestyle habits better than MetS. To answer this question, We utilized data on the population from the National Health and Nutrition Examination Survey (NHANES) to build machine learning (ML) models. Then, we employed publicly available genetic data within the Mendelian randomization (MR) analysis framework to assess the evidence linking the components of CircS to psoriasis in terms of causal relationships.

## 2 Materials and methods

**Figure 1:**
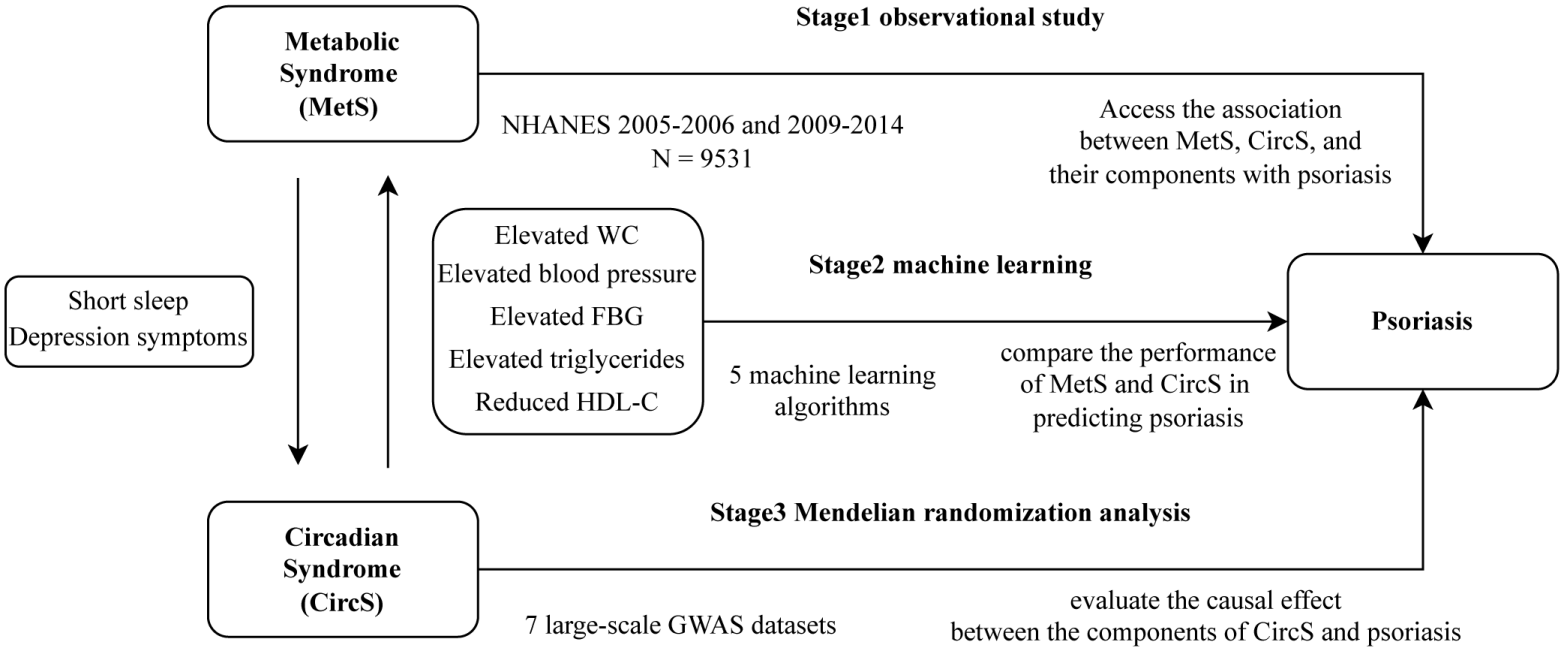
Overall study design of the present study.

Our study was conducted in three stages in **Figure 1**. In the first stage, we conducted three multiple logistic regression between MetS, CircS, and their components with psoriasis using data from the NHANES database to explore their association. In the second stage, we select variables from the first stage and use five ML algorithms to compare the performance of MetS and CircS in predicting psoriasis. Select the best-performing model for interpretation and calculate the importance of each variable in the prediction. In the final stage, we used MR analyses of summary statistics from a genome-wide association study (GWAS) to evaluate the causal effect between the components of CircS and psoriasis.

### 2.1 Observational study

#### 2.1.1 Data sources and study population

NHANES is a cross-sectional survey updated biennially by the Centers for Disease Control and Prevention to assess adults’ and children’s health and nutritional status in the United States and track changes over time. Questionnaires, physical examinations, and lab work make up the survey. NHANES provides extensive data that accurately represents the US civilian and noninstitutionalized population through a stratified, clustered, and multistage probability sampling technique that selects from cities, blocks, and households. This survey was approved by the Ethics Review Board of the National Center for Health Statistics, and all participants signed written informed consent.

We enrolled 40,816 participants from four survey cycles (2005–2006, 2009–2010, 2011–2012, 2013–2014). The exclusion criteria included: (a) Participants under the age of 20 years; (b) Participants with missing laboratory test data or questionnaires to define MetS and CircS; (c) Participants with pregnant. The final sample for subsequent analysis comprised 9,531 participants. We employed multiple imputations by chained equations with four replications to impute the remaining missing data (15).

#### 2.1.2 Exposure variable: MetS and CircS

According to the criteria published in 2009 by the International Diabetes Federation Task Force on Epidemiology and Prevention; the National Heart, Lung, and Blood Institute; the American Heart Association; and others (16), MetS was diagnosed at least three of the following: (1) elevated waist circumference (WC) (≥102 cm for males, ≥88 cm for females); (2) elevated blood pressure (systolic ≥130 mmHg or diastolic ≥85 mmHg or both) or drug treatment for hypertension; (3) elevated fasting blood glucose (FBG) (≥100 mg/dL) or drug treatment for elevated FBG; (4) reduced High density lipoprotein cholesterol (HDL-C) (<40 mg/dL for males,<50 mg/dL for females) or drug treatment for reduced HDL-C; (5) elevated triglycerides (TG) (≥150 mg/dL) or drug treatment for elevated TG.

CircS consisted of seven components: reduced sleep duration, depression symptoms, and the five components of MetS. Participants with ≥4 of the abovementioned components were considered to have CircS.

Self-reported sleep duration<6h/day was considered short sleep. Depression symptoms were defined by the patient health status questionnaire-9 (PHQ-9) (17). The score of PHQ-9 in 5–9, 10–14, 15–19 and ≥20 represent mild, moderate, moderately severe, and severe depression. Participants with the scores of PHQ-9 ≥5 were considered to have depression symptoms.

#### 2.1.3 Outcome variable: psoriasis

Psoriasis was established by confirming a positive answer to the question, “Have you ever been told by a healthcare provider that you had psoriasis?”

#### 2.1.4 Covariates collection

We included age, gender, race, educational level, family income, smoking status, and drinking status as covariates based on the literature (18). Specifically, age was categorized into three stages: 20–39, 40–59 and ≥60 years. Races included Mexican American, Other Hispanic, non-Hispanic white, non-Hispanic black, and other races. Family income was categorized into three classes based on the family poverty income ratio: low income (≤1.3), middle income (1.3–3.5), and high income (>3.5). The education level was classified into high school graduate or less, some college, and college graduate or above. We categorize participants into three smoking statuses: never (<100 cigarettes in a lifetime), former (≥100 cigarettes in a lifetime and smoking not at all now), and now (≥100 cigarettes in a lifetime and smoking some days or every day now). Drinking ≥12 drinks per year is considered drinking status.

#### 2.1.5 Statistical analysis

We performed weighting based on the weights recommended by the NHANES analysis guidelines: the sampling weight for each cycle is equal to the 2-year MEC weight of the fasting subsample/4. Participants’ continuous variables were characterized using mean values, while categorical variables were described using percentage frequencies. Use student t-test to compare differences in continuity variables between MetS or CircS groups that follow a normal distribution; Use the Mann-Whitney U test for skewed distribution data. Categorical variables were compared using the chi-square test. We built three multiple logistic models to examine the association between CircS or MetS with psoriasis. In the non-adjusted model, no factor was adjusted. In the minimally adjusted model, age, gender, and race were adjusted. In the fully adjusted model, age, gender, race, educational level, family income, smoking status, and drinking status were all adjusted. On that basis, we further analyzed the correlation between the components of circs and psoriasis. All statistical analyses were performed in the R environment (version 4.3.2). We calculated the odds ratio (OR) and 95% confidence interval (CI), and *P*< 0.05 (two-sided) was defined as the threshold for statistical significance.

### 2.2 Machine learning

#### 2.2.1 ML model strategies

To compare the predictive value of CircS and MetS for psoriasis, we incorporated all covariates and each component of CircS or MetS in the ML dataset. Five ML algorithms, k-nearest neighbor classification (KNN), support vector machine (SVM), eXtreme Gradient Boosting (XGBoost), Light Gradient Boosting Machine (LightGBM), Categorical Features+Gradient Boosting (CatBoost) are used for model construction. The dataset was randomly split into two disjoint sets: a training set (80% of the total data) and a test set (the remaining 20% of the data not in the training set) using the Pareto principle (19). In the training dataset, we used Bayesian optimization with a fivefold cross-validation to perform automatic hyperparameter tuning for each model. For the purpose of testing the training models, we used the testing sets to compare the performance of CircS and MetS models. The following performance metrics were used: $Precision=\;\frac{TP}{TP+FN}$ $Recall=\;\frac{TP}{TP+FP}$ $f1-Score=2\times \frac{Precision\times Recall}{Precision+Recall}$ . TP indicates a true positive where the model predicts that the patient has psoriasis and the patient actually has the disease. FP represents a false positive, and FN denotes a false negative. Due to data imbalance in the dataset, the F1 score and the area under the precision-recall curve (AUPRC) better summarize biomarkers and reflect model performance (20). All ML models were built with the “mlr3verse” package. The “modEVA 3.11” package was utilized to calculate the F1 score and AUPRC.

#### 2.2.2 Explainable ML model

After summarizing the F1 score and AUPRC for each model, we selected the most suitable model for identifying and interpreting the disease. SHapley Additive exPlanations (SHAP) is a method to explain ML models and address their black-box issue (21). Shapley values originate from the cooperative game theory field, which can be used to assess the interaction/joint effect of two features and calculate the importance of a feature by quantifying the marginal impact (22). Shapviz (version 0.9.2) package was used to give local explanations and allow computation of the contribution of each variable to each individual’s prediction by Shapley values. We use SHAP beeswarm plots to visualize the results of the selected models.

### 2.3 Mendelian randomization

#### 2.3.1 Basic concept of MR analysis

Mendelian randomization (MR) is a method using measured genetic variants (single nucleotide polymorphisms [SNPs]) as instrumental variables (IV) to examine an exposure’s causal effect on an outcome (23). Compared to traditional observational methods, MR analysis is less vulnerable to bias from reverse causation and confounding. We performed a two-sample MR analysis to validate the association of each component of circs with psoriasis. Furthermore, a reverse MR analysis was performed to avoid any chance of reverse causality.

#### 2.3.2 Data sources

Our MR analyses used publicly available summary statistics from large-scale GWAS datasets. Data for WC and hypertension were obtained from MRC-IEU UK Biobank OpenGWAS (24), which included 462,166 and 462,933 European individuals. Genetic variants associated with FBG (25) and depression symptoms (26) were extracted from a joint meta-analysis of GWAS in 58,074 and 807,553 individuals, respectively. The summary level exposure datasets for triglycerides (27), HDL-C (28), and short sleep (29) were extracted from UKBiobank by three different research groups. As to psoriasis (9,267 cases, 364,071 controls), the summary statistics were obtained from the FinnGen consortium’s R9 release of GWAS data (30). All subjects were of European descent. Age, sex, and other major variables were taken into account during the analyses of these data. Specific information is listed in **Supplementary Table 1.**

#### 2.3.3 Selection of SNPs for MR analysis

The genetic variants used for this MR analyses need to satisfy three assumptions: (1) the genetic variants must be strongly correlated with the components of CircS; (2) the genetic variant is independent of any potential confounders between CircS and psoriasis; (3) the genetic variants affect the psoriasis only through the components of CircS. To fulfill these three assumptions, we used several quality assurance procedures for IV selection: (1) Candidate IVs were picked from SNPs associated with the components of CircS at the locus-wide significance threshold of *P*< 5 × 10^-8^. Clumping parameters included a linkage disequilibrium (LD) threshold of *R* ^2^< 0.01 and a window size of 10,000 kb. The LD value was calculated using the European-based 1,000 Genome Project (31) as a reference, and SNPs that did not meet the requirements were removed; (2) we used the PhenoScanner GWAS database (32) to verify all selected SNPs and removed SNPs associated significantly with any potential confounder at the genome-wide level; (3) the remaining SNPs were extracted from the psoriasis datasets and calculated F-statistics (33) for each SNP to quantify the strength of the psoriasis. IVs with F*-*values< 10 were regarded as weak and were excluded.

#### 2.3.4 MR analysis

We chose four MR methods, including inverse variance weighted (IVW), MR-Egger, weighted median (WM), and maximum likelihood (ML) for two-sample MR analyses. For SNPs without horizontal pleiotropy, the IVW method is the primary tool for calculating causal effect values to produce unbiased estimates. The remaining three methods were used as secondary methods to correct for pleiotropy. Several sensitivity analyses were conducted to assess whether this analysis violates the MR assumptions and determine the results’ reliability and validity. Cochrane’s *Q* test was applied to ascertain the heterogeneity among SNPs associated with each component of CircS. If there was heterogeneity, we used a random-effect IVW model; otherwise, the fixed-effect IVW model was performed (34). We ran MR-Egger regressions and MR-PRESSO analyses to rule out potential pleiotropy. Finally, a leave-one-out approach was used to examine the possible impacts of pleiotropic SNPs on the causal estimates by repeating analyses after removing one SNP at a time. We used TwoSampleMR (version 0.5.8) (35) and MRPRESSO (version 1.0) packages for MR and sensitivity analysis.

## 3 Results

### 3.1 Baseline characteristics of NHANES

A total of 9,531 participants were eligible and divided into four groups by MetS and CircS in these four cycles. As shown in **Table 1**, most baseline characteristics showed significant differences between the groups. Participants with MetS or CircS were older (MetS: 57.0 [45.0;68.0]; CircS: 56.0 [45.0;67.0]) and more likely to be smoking (MetS: 50%; CircS: 51.3%) status. Psoriasis was more common in both MetS (3.76%) and CircS (3.88%) and more prevalent among CircS participants. By definition, CircS participants differed more from MetS participants regarding sleep duration and depression symptoms. Specifically, participants in CircS had less sleep (MetS: 41.8%; CircS: 56.8%) and were more depressed (MetS: 30.8%; CircS: 43.7%) than those in MetS. In particular, among depressive symptoms, the CircS group was more severe than the MetS group in all phases. We also tested the reliability of some of the covariates after multiple imputations. There were no statistical differences among covariates in 4 replications (**Supplementary Table 2**).

**Table 1:** Characteristics of participants by categories of MetS and CircS: NHANES 2005–2006 and 2009–2014.

Variables	All (n=9531)	MetS group		*P*	CircS group		P
		MetS (n=4338)	Non-MetS (n=5193)		CircS (n=3634)	Non-CircS (n=5897)	
Age (mean (SD))	50.0 [36.0;63.0]	57.0 [45.0;68.0]	43.0 [30.0;56.0]	<0.001	56.0 [45.0;67.0]	44.0 [31.0;60.0]	<0.001
Age group				<0.001			<0.001
20–39	2978 (31.2%)	704 (16.2%)	2274 (43.8%)		594 (16.3%)	2384 (40.4%)	
40–69	3494 (36.7%)	1664 (38.4%)	1830 (35.2%)		1462 (40.2%)	2032 (34.5%)	
60+	3059 (32.1%)	1970 (45.4%)	1089 (21.0%)		1578 (43.4%)	1481 (25.1%)	
Gender				0.023			<0.001
Male	4741 (49.7%)	2102 (48.5%)	2639 (50.8%)		1683 (46.3%)	3058 (51.9%)	
Female	4790 (50.3%)	2236 (51.5%)	2554 (49.2%)		1951 (53.7%)	2839 (48.1%)	
Race				<0.001			<0.001
Mexican American	1473 (15.5%)	707 (16.3%)	766 (14.8%)		598 (16.5%)	875 (14.8%)	
Other Hispanic	892 (9.36%)	408 (9.41%)	484 (9.32%)		351 (9.66%)	541 (9.17%)	
Non-Hispanic White	4202 (44.1%)	1956 (45.1%)	2246 (43.3%)		1585 (43.6%)	2617 (44.4%)	
Non-Hispanic Black	2003 (21.0%)	937 (21.6%)	1066 (20.5%)		830 (22.8%)	1173 (19.9%)	
Other Race	961 (10.1%)	330 (7.61%)	631 (12.2%)		270 (7.43%)	691 (11.7%)	
Income to poverty ratio				<0.001			<0.001
Low	3137 (32.9%)	1562 (36.0%)	1575 (30.3%)		1396 (38.4%)	1741 (29.5%)	
Moderate	3498 (36.7%)	1602 (36.9%)	1896 (36.5%)		1311 (36.1%)	2187 (37.1%)	
High	2896 (30.4%)	1174 (27.1%)	1722 (33.2%)		927 (25.5%)	1969 (33.4%)	
Education				<0.001			<0.001
High school or less	4463 (46.8%)	2289 (52.8%)	2174 (41.9%)		1940 (53.4%)	2523 (42.8%)	
Some college	2874 (30.2%)	1336 (30.8%)	1538 (29.6%)		1134 (31.2%)	1740 (29.5%)	
College graduate or higher	2194 (23.0%)	713 (16.4%)	1481 (28.5%)		560 (15.4%)	1634 (27.7%)	
Smoking status				<0.001			<0.001
Never	5188 (54.4%)	2169 (50.0%)	3019 (58.1%)		1772 (48.8%)	3416 (57.9%)	
Former	2383 (25.0%)	1283 (29.6%)	1100 (21.2%)		1049 (28.9%)	1334 (22.6%)	
Now	1960 (20.6%)	886 (20.4%)	1074 (20.7%)		813 (22.4%)	1147 (19.5%)	
Drinking status				<0.001			<0.001
No	4546 (47.7%)	2242 (51.7%)	2304 (44.4%)		1886 (51.9%)	2660 (45.1%)	
Yes	4985 (52.3%)	2096 (48.3%)	2889 (55.6%)		1748 (48.1%)	3237 (54.9%)	
BMI (kg/m2) (mean (SD))	29.5 (6.95)	33.1 (6.88)	26.5 (5.45)	<0.001	33.3 (7.06)	27.2 (5.75)	<0.001
WC	100 (16.7)	110 (14.8)	92.1 (13.3)	<0.001	111 (15.2)	94.1 (14.3)	<0.001
Triglycerides	110 [74.0;171]	165 [110;237]	85.0 [61.0;116]	<0.001	171 [115;245]	89.0 [64.0;124]	<0.001
Direct HDL-C	50.0 [42.0;61.0]	44.0 [37.0;52.0]	56.0 [48.0;66.0]	<0.001	43.0 [37.0;52.0]	55.0 [46.0;65.0]	<0.001
Plasma glucose	101 [93.0;116]	114 [102;141]	95.0 [89.0;101]	<0.001	114 [102;143]	96.0 [90.0;104]	<0.001
Systolic pressure	121 [111;133]	127 [117;139]	116 [108;126]	<0.001	127 [116;139]	117 [109;128]	<0.001
Diastolic pressure	69.3 [62.0;76.7]	71.3 [62.7;79.3]	68.7 [62.0;74.7]	<0.001	71.3 [63.3;79.3]	68.7 [62.0;75.3]	<0.001
Sleep time	7.00 [6.00;8.00]	7.00 [6.00;8.00]	7.00 [6.00;8.00]	0.169	6.00 [5.00;8.00]	7.00 [6.00;8.00]	<0.001
Elevated WC				<0.001			<0.001
No	3913 (41.1%)	503 (11.6%)	3410 (65.7%)		427 (11.8%)	3486 (59.1%)	
Yes	5618 (58.9%)	3835 (88.4%)	1783 (34.3%)		3207 (88.2%)	2411 (40.9%)	
Elevated blood pressure				<0.001			<0.001
No	4444 (46.6%)	802 (18.5%)	3642 (70.1%)		659 (18.1%)	3785 (64.2%)	
Yes	5087 (53.4%)	3536 (81.5%)	1551 (29.9%)		2975 (81.9%)	2112 (35.8%)	
Elevated FBG				<0.001			<0.001
No	4291 (45.0%)	620 (14.3%)	3671 (70.7%)		549 (15.1%)	3742 (63.5%)	
Yes	5240 (55.0%)	3718 (85.7%)	1522 (29.3%)		3085 (84.9%)	2155 (36.5%)	
Elevated triglycerides				<0.001			<0.001
No	6432 (67.5%)	1750 (40.3%)	4682 (90.2%)		1338 (36.8%)	5094 (86.4%)	
Yes	3099 (32.5%)	2588 (59.7%)	511 (9.84%)		2296 (63.2%)	803 (13.6%)	
Reduced HDL-C				<0.001			<0.001
No	6573 (69.0%)	1982 (45.7%)	4591 (88.4%)		1528 (42.0%)	5045 (85.6%)	
Yes	2958 (31.0%)	2356 (54.3%)	602 (11.6%)		2106 (58.0%)	852 (14.4%)	
Sleep ≤ 6 h/day				0.001			<0.001
No	5720 (60.0%)	2526 (58.2%)	3194 (61.5%)		1570 (43.2%)	4150 (70.4%)	
Yes	3811 (40.0%)	1812 (41.8%)	1999 (38.5%)		2064 (56.8%)	1747 (29.6%)	
Depression symptoms				<0.001			<0.001
None-minimal	7122 (74.7%)	3002 (69.2%)	4120 (79.3%)		2046 (56.3%)	5076 (86.1%)	
Mild	1514 (15.9%)	775 (17.9%)	739 (14.2%)		940 (25.9%)	574 (9.73%)	
Moderate	543 (5.70%)	326 (7.51%)	217 (4.18%)		388 (10.7%)	155 (2.63%)	
Moderately Severe	247 (2.59%)	162 (3.73%)	85 (1.64%)		182 (5.01%)	65 (1.10%)	
Severe	105 (1.10%)	73 (1.68%)	32 (0.62%)		78 (2.15%)	27 (0.46%)	
Psoriasis				<0.001			<0.001
No	9243 (97.0%)	4175 (96.2%)	5068 (97.6%)		3493 (96.1%)	5750 (97.5%)	
Yes	288 (3.02%)	163 (3.76%)	125 (2.41%)		141 (3.88%)	147 (2.49%)	

Use student t-test for normal distribution data and the Mann-Whitney U test for skewed distribution data in continuity variables. Categorical variables were compared using the chi-square test. MetS, Metabolic Syndrome; CircS, Circadian Syndrome; BMI, body mass index; WC, waist circumference; HDL-C, high density lipoprotein cholesterol; FBG, fasting blood glucose

### 3.2 Multivariate regression analysis

**Figure 2:**
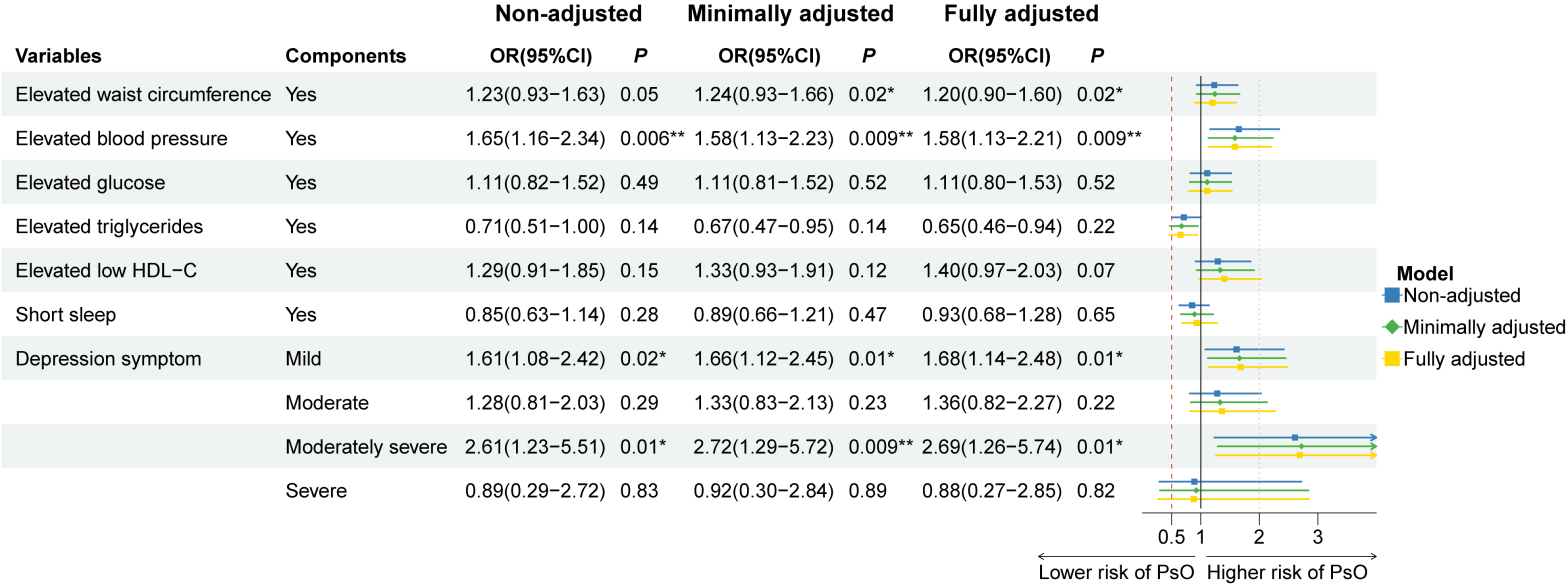
Association of the components of circadian syndrome with the prevalence of psoriasis in three multiple logistic regression models. OR: odds ratio; CI: confidence interval. * *P*< 0.05, ** *P*< 0.01. Non-adjusted model adjusted no factor. Minimally adjusted model adjusted age, gender, and race. Fully adjusted model adjusted age, gender, race, educational level, family income, smoking status, and drinking status.

Sample-weighted multiple regression analyses in **Table 2** showed a positive relationship between CircS with psoriasis prevalence in the non-adjusted model (OR = 1.48, 95%CI: 1.09–1.99, *P* = 0.012), the minimally adjusted model (OR = 1.40, 95%CI: 1.03–1.91, *P* = 0.033) and the fully adjusted model (OR = 1.40, 95%CI: 1.02–1.91, *P* = 0.039). There was also a positive correlation between MetS and psoriasis, with OR of 1.59 (95%CI: 1.12–2.26, *P* = 0.01), 1.48 (95%CI: 1.04–2.11, *P* = 0.031), and 1.53 (95%CI: 1.07–2.17, *P* = 0.02) for prevalent psoriasis in the three models, respectively. These three models were further employed to investigate the association between the CircS components with psoriasis in **Figure 2**. Psoriasis prevalence was found to be promoted by elevated blood pressure (OR_non-adjusted_ = 1.65, *P*_non-adjusted_ = 0.006; OR_minimally adjusted_ = 1.58, *P*_minimally adjusted_ = 0.009; OR_fully adjusted_ = 1.58, *P*_fully adjusted_ = 0.009) and depression symptoms in all three models. The OR of depression symptoms was greater in moderately severe (OR_non-adjusted_ = 2.61, *P*_non-adjusted_ = 0.01; OR_minimally adjusted_ = 2.72, *P*_minimally adjusted_ = 0.009; OR_fully adjusted_ = 2.69, *P*_fully adjusted_ = 0.01) than in mild (OR_non-adjusted_ = 1.61, *P*_non-adjusted_ = 0.02; OR_minimally adjusted_ = 1.66, *P*_minimally adjusted_ = 0.01; OR_fully adjusted_ = 1.68, *P*_fully adjusted_ = 0.01). In the minimally and fully adjusted models, the risk of psoriasis was 1.24 and 1.20 times greater in those with elevated WC, respectively, with *P* of 0.024 and 0.023, compared to those without. The remaining components showed no statistically significant association with the prevalent psoriasis.

**Table 2:** Association of MetS and CircS with the prevalence rates of psoriasis.

Variables	Non-adjusted model*		Minimally adjusted model**		Fully adjusted model***	
	OR (95%CI)	P	OR (95%CI)	P	OR (95%CI)	P
Circadian syndrome						
No	Ref		Ref		Ref	
Yes	1.48(1.09–1.99)	0.012	1.40(1.03–1.91)	0.033	1.40(1.02–1.91)	0.039
Metabolic syndrome						
No	Ref		Ref		Ref	
Yes	1.59(1.12–2.26)	0.010	1.48(1.04–2.11)	0.031	1.53(1.07–2.17)	0.020

*Non-adjusted model adjusted no factor. **Minimally adjusted model adjusted age, gender, and race. ***Fully adjusted model adjusted age, gender, race, educational level, family income, smoking status, and drinking status. OR, odds ratio; CI, confidence interval.

### 3.3 Model performance comparison

**Figure 3:**
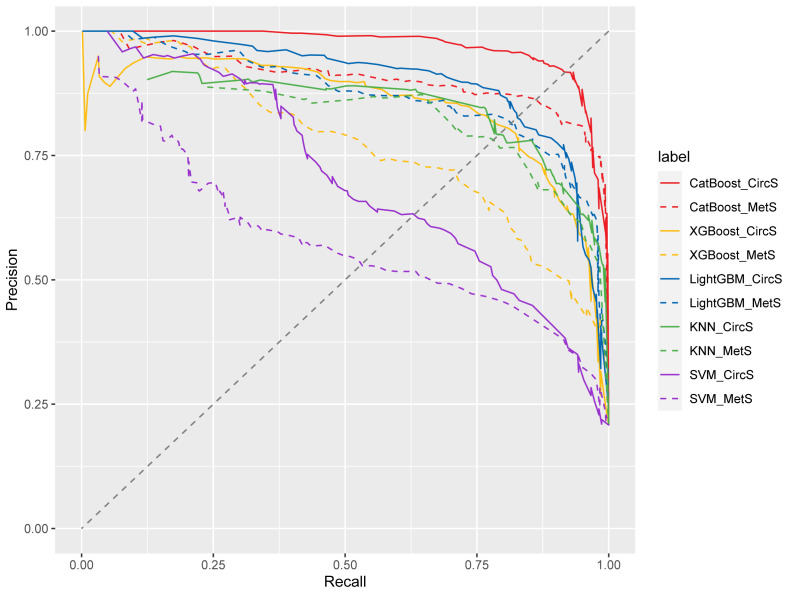
Precision-recall curve from five models for circadian syndrome (CircS) and Metabolic Syndrome (MetS) in predicting psoriasis. KNN, k-nearest neighbor classification; SVM, support vector machine; XGBoost, eXtreme Gradient Boosting; LightGBM, Light Gradient Boosting Machine; CatBoost, Categorical Features+Gradient Boosting.

We generated five algorithms to predict the probability of psoriasis using each MetS or CircS component, respectively. **Figure 3** displays the AUPRC curves of the five models on the test set to demonstrate their discriminant performance. Among the five models in CircS, CatBoost (AUPRC = 0.969) has the best prediction effect on psoriasis, followed by LightGBM (AUPRC= 0.901), XGBoost (AUPRC = 0.85), KNN (AUPRC = 0.735), and SVM (AUPRC = 0.704). Predicting psoriasis using MetS had similar results, with CatBoost (AUPRC = 0.907) having the best performance, followed by LightGBM (AUPRC= 0.873), XGBoost (AUPRC = 0.772), KNN (AUPRC = 0.608), and SVM (AUPRC = 0.582). Comparing the performance of CircS components and MetS components in each algorithm, we find that CircS components outperform MetS components in every algorithm. Components of CircS are better predictors of psoriasis in these algorithms. The recall, precision, F1 scores, AUPRC, and 95%CI of AUPRC for all five ML models are shown in **Table 3**. **Supplementary Table 3** shows the differences between AUPRC compared pairwise for all models. The 95% bootstrap CI for this difference was calculated. Each 95% bootstrap CI does not include 0, implying that there are differences in each of the AUPRCs being compared.

**Table 3:** Model performance metrics.

Models	Recall	Precision	F1	AUPRC	95% CI
CatBoost_CircS	0.908	0.925	0.916	0.969	0.952–0.981
CatBoost_MetS	0.857	0.864	0.861	0.907	0.880–0.927
XGBoost_CircS	0.719	0.851	0.78	0.852	0.821–0.878
XGBoost_MetS	0.54	0.768	0.634	0.772	0.736–0.803
LightGBM_CircS	0.795	0.871	0.831	0.901	0.875–0.923
LightGBM_MetS	0.726	0.829	0.774	0.873	0.844–0.897
KNN_CircS	0.853	0.78	0.815	0.846	0.815–0.873
KNN_MetS	0.729	0.789	0.758	0.819	0.786–0.848
SVM_CircS	0.452	0.727	0.647	0.704	0.665–0.738
SVM_MetS	0.334	0.609	0.586	0.582	0.543–0.622

CircS, Circadian Syndrome; MetS, Metabolic Syndrome; AUPRC, area under the precision-recall curve; CI, confidence interval; KNN, k-nearest neighbor classification; SVM, support vector machine; XGBoost, eXtreme Gradient Boosting; LightGBM, Light Gradient Boosting Machine; CatBoost, Categorical Features+Gradient Boosting

### 3.4 Visualization of feature importance

**Figure 4:**
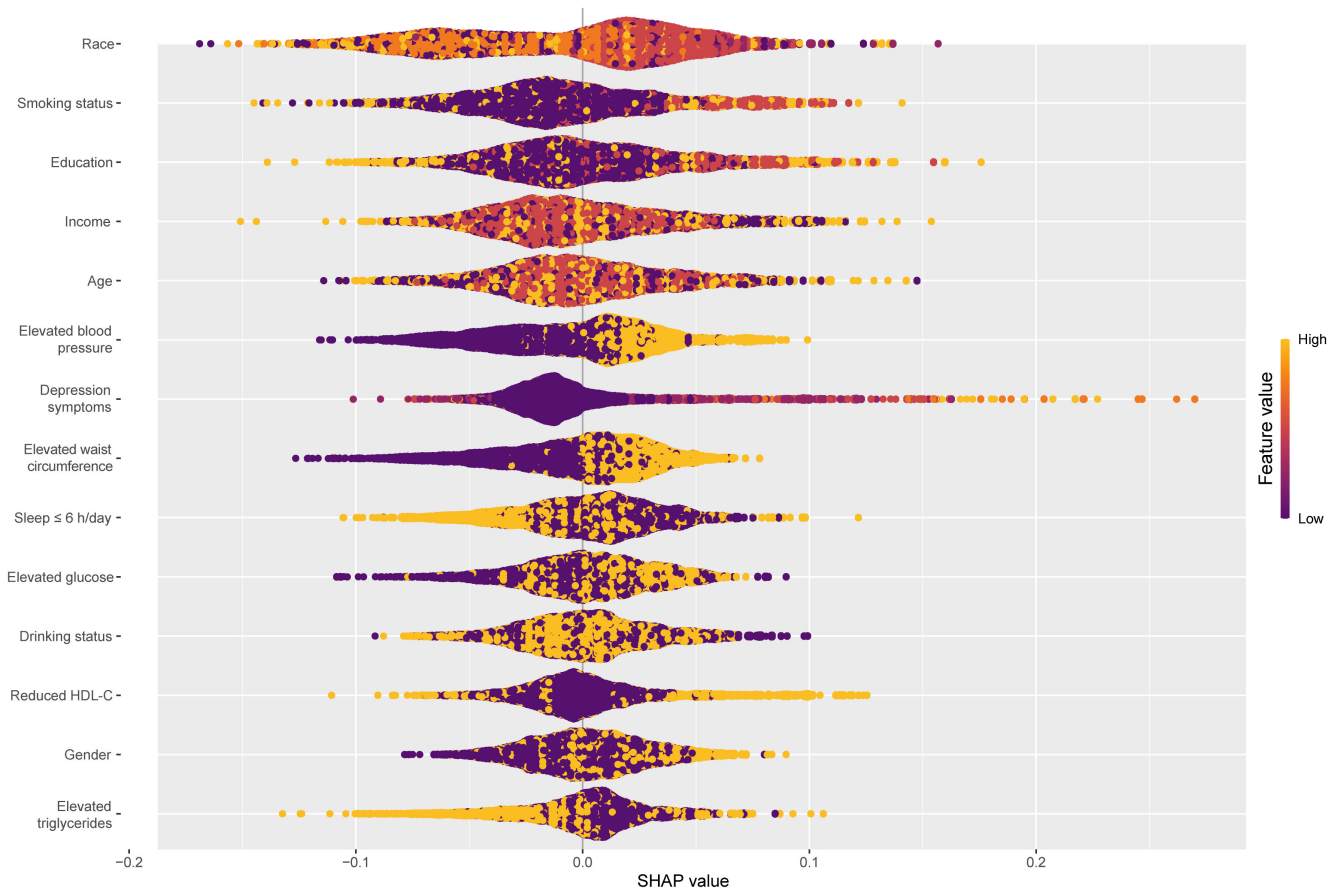
Global explainability beeswarm plots of the CatBoost model for predicting psoriasis by each component of CircS. CatBoost, Categorical Features+Gradient Boosting; CircS, Circadian Syndrome.

Combined with **Table 3** and **Supplementary Table 3**, each component of circs using the CatBoost algorithm predicted psoriasis performance best. We perform SHAP model interpretation and feature importance visualization for this model. The SHAP variable importance and beeswarm plot (**Figure 4**) present the comprehensive impact of each feature on psoriasis, with features arranged in descending order based on their significance. A positive SHAP value signifies a positive correlation between the value of a feature and the prevalence of psoriasis. Larger values contribute more to the prediction of psoriasis. Among the components of CircS, elevated blood pressure, depression symptoms, elevated WC, and short sleep contributed more to the prediction of psoriasis. Elevated blood pressure, depression symptoms, and elevated WC were positively associated with the prevalence of psoriasis, whereas short sleep was not significantly different in the figure.

### 3.5 MR of CircS and psoriasis

**Figure 5:**
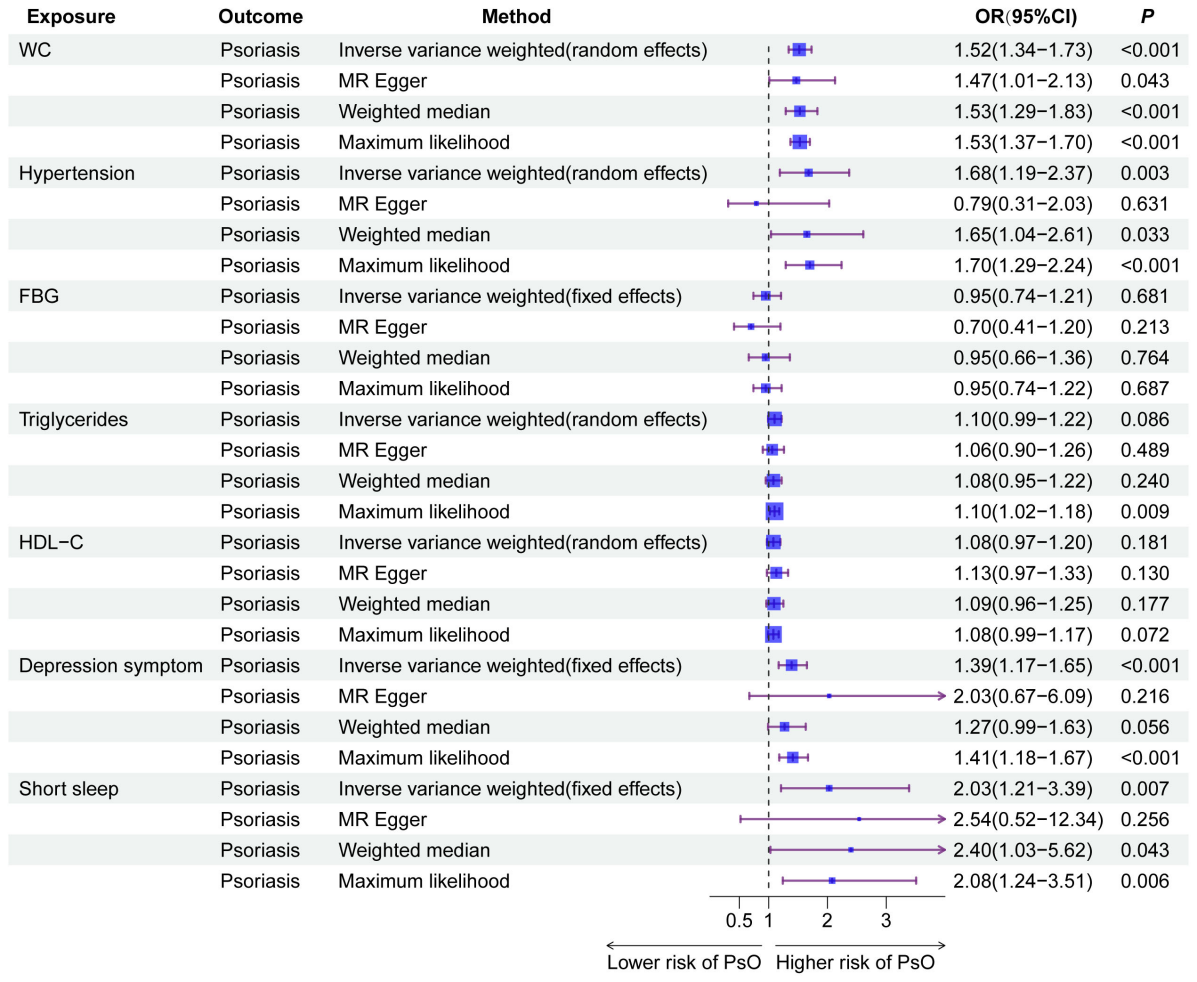
The results of Mendelian randomization analyses for the causal association between the components of CircS and MetS with psoriasis. CircS, Circadian Syndrome; MetS, Metabolic Syndrome.

After filtering according to the above selection standard, the number of IVs for WC, hypertension, FBG, triglycerides, HDL-C, depression symptoms, and short sleep were 456; 252; 18; 334; 50; 41; and 42, respectively. Abnormal IVs were removed through MR-PRESSO global outlier test, and F-statistics for all IVs exceeded 10, ranging from 28.77 to 2059.43 (**Supplementary Table 4**). Cochran’s Q test in **Supplementary Table 5** indicated significant heterogeneity in WC, hypertension, triglycerides, and HDL-C. Therefore, a multiplicative random-effects model was utilized for these four IVW analyses. Under the IVW model in **Figure 5**, there were asignificant causal relationships between WC (OR = 1.52, 95%CI: 1.34–1.73, *P* = 1.35e^-10^), hypertension (OR = 1.68, 95%CI: 1.19–2.37, *P* = 0.003), depression symptoms (OR = 1.39, 95%CI: 1.17–1.65, *P* = 1.51e^-4^), and short sleep (OR = 2.03, 95%CI: 1.21–3.39, *P* = 0.007) with psoriasis risk, while no causal relationship was found for FBG, triglycerides, and HDL-C. The remaining three methods further validated the results. The sensitivity analysis results showed that the p-value of MR-Egger intercept between the selected instruments was less than 0.05 (**Supplementary Table 5**), which indicates that the associations were not affected by significant horizontal pleiotropy. As determined by the leave-one-out analyses, no single SNP significantly affected the results for each component of CircS, ensuring the reliability of the results (**Supplementary Figure 1**). Moreover, the results of reverse MR analyses indicate no reverse associations of genetic susceptibility to the components of CircS with psoriasis (**Supplementary Table 6**).

## 4 Discussion

This cross-sectional study examined the link between MetS, CircS, and their components with psoriasis risk in US adults. It also compared the predictive power of CircS and MetS in identifying psoriasis via machine learning. Our results revealed that CircS and its components were positively associated with the prevalence of psoriasis. CircS was a better predictor for prevalent psoriasis than Mets, with elevated blood pressure, depression symptoms, and elevated WC contributing more to the prediction. These results were further confirmed in MR analyses.

Similar to the majority of literature on MetS as an intrinsic risk factor for psoriasis (36), we found that MetS increases the prevalence of psoriasis. Combining the effects of sleep disorders and depression on psoriasis, we noted the new concept of CircS. The circadian system regulates a wide range of bodily processes, including gene expression, hormone release, and energy expenditure, among many other critical physical processes, and is essential to maintaining human health and metabolism (37). Circadian rhythms play a crucial part in glucose and insulin regulation (38). Several studies have found that imbalances in circadian rhythms not only lead to metabolic disorders such as obesity but also exacerbate depression symptoms (39, 40), which explains the increase of depression in MetS. CircS provides a better explanation of the MetS itself and its comorbidities, such as sleep disturbances and depression. Thus, CircS may better cover the symptoms of psoriasis patients. As we speculate, CircS was positively associated with psoriasis in this study, with a higher proportion of CircS participants having psoriasis and better performance predicting psoriasis than MetS.

Elevated blood pressure, depression symptoms, elevated WC, and short sleep were more important in predicting psoriasis in the optimal ML model as interpreted by SHAP. The MR analyses further verified the causality of these results. Unfortunately, logistic regression did not show significance for short sleep. Elevated blood pressure and elevated WC are components shared by MetS and CircS. In a large national cohort study totaling 256,356 participants over 11 years, psoriasis incidence was higher in participants with hypertension, and the results were still significant after adjustment for confounders (41). Hypertension and psoriasis may be related to a common inflammatory pathway. Inflammation contributes to the development of hypertension through a complex interplay involving immune cells, cytokines, and oxidative stress (42). Accumulating evidence suggests that low-grade chronic inflammation plays a significant role in the initiation and maintenance of essential hypertension (43). Long-term stimulation by chronic inflammation can lead to vascular remodeling and endothelial dysfunction, promote immune cell activation, and accumulate reactive oxygen species (ROS), contributing to elevated blood pressure (44). Psoriasis is similarly afflicted by ROS, associated with increased ROS production and decreased antioxidant potential, leading to oxidative stress and damage to cell components (45). ROS is a significant factor in the development and progression of hypertension and psoriasis, contributing to the chronic inflammatory processes and immune dysregulation associated with the condition.

Several epidemiological investigations have confirmed that obesity is a risk factor for psoriasis, especially centripetal obesity characterized by elevated WC (46, 47). White adipose tissue (WAT) expansion is central to obesity, responding to caloric surplus through unhealthy expansion, leading to obesity-related complications (48). The expansion of WAT in obesity is accompanied by inflammation of the adipose tissue, which eventually develops into chronic low-grade systemic inflammation. In addition to the chronic inflammatory state of obesity, the adipokine leptin, which regulates energy balance and metabolism in adipose tissue, has been implicated in developing psoriasis. It is highly expressed in psoriasis skin lesions and serum (49). These inflammatory responses involving various cytokines, chemokines, and hormones secreted by different types of adipose tissue cells ultimately lead to an immune disorder similar to psoriasis.

Depression symptoms and short sleep are components unique to CircS. Our results indicate that depression symptoms were a strong predictor of psoriasis, with an increased risk as the severity of the depression symptoms worsened. Depression was found to be independently associated with an increased risk of psoriasis in a large prospective cohort of US women (50). Two MR studies found the same results (51, 52). It has recently been discovered that depression has an inflammatory pattern similar to psoriasis. Overactivation of microglia in a depressed mouse model leads to differentiation of T cells into Th17 cells, an increase in cytokines such as IL-17 and IL-6, and a decrease in Treg. When the model mice were treated with IL-17A, the level of inflammation was equalized, IL-10 was upregulated, and the number of Treg increased. The depression symptoms of mice gradually alleviate (53). However, the relationship between depression and psoriasis still lacks support from high-quality randomized controlled trials. Although short sleep had a causal effect on psoriasis in MR and contributed to the prediction of psoriasis, the results of SHAP visualization and logistic regression were not significant. Short sleep and psoriasis can also be linked through inflammatory networks, particularly tumor necrosis factor-α and IL-6, related to psoriasis pathogenesis and sleep regulation (54).

The present study has several strengths. Firstly, our study exploring the relationship between MetS and CircS with psoriasis had a relatively large sample size. Logistic regression results combined with MR analyses clarified the direction of causality, and the results were made more robust by avoiding confounding factors. Secondly, We use five ML algorithms to compare the performance of MetS and CircS for predicting psoriasis and explain the best-performing model. As far as we know, no research has been conducted on the correlation between CircS and the prevalence of psoriasis. The study also has several limitations. First, the cross-sectional design meant that variables could not be extracted strictly to our specific needs, such as psoriasis and sleep duration were self-reported rather than obtained by diagnosis and precise measurement. We defined participants with PHQ-9 scores greater than 5 as depression symptoms and did not use mild depression with PHQ-9 scores greater than 10, which better summarizes the psychological state of anxiety or depression. At the same time, the cycles included in the study lacked surveys on taking medication for depression due to the different questionnaires for each cycle. Second, in contrast to the cross-sectional study on a multiethnic US population, our MR analyses focused on individuals of European ancestry. Individual ethnic studies are also needed to eliminate potential confounding by heterogeneity across populations. Third, although we performed MR analyses between the components of CircS and psoriasis to test their respective causal effects, we still had difficulty determining the direction of CircS and psoriasis due to the lack of a GWAS database for CircS.

## 5 Conclusion

To summarize, both the MetS and CircS were positively associated with psoriasis in NHANES data from 4 cycles. Moreover, CircS demonstrated superior predictive ability for prevalent psoriasis compared to MetS, with elevated blood pressure, depression symptoms, and elevated WC contributing more to the prediction. We should keep a close watch on the risk of psoriasis with CircS in the presence of these three components. The introduction of the concept of CircS has refined risk management in psoriasis.

## Data availability statement

The original contributions presented in the study are included in the article/**Supplementary Material**. Further inquiries can be directed to the corresponding authors.

## Author contributions

YG: Conceptualization, Software, Writing – original draft, Writing – review & editing. XY: Resources, Writing – original draft. WTZ: Software, Visualization, Writing – original draft. SH: Data curation, Resources, Writing – original draft. WMZ: Resources, Validation, Writing – original draft. XZ: Conceptualization, Writing – original draft, Writing – review & editing.
