# Supplementary material for: The circadian syndrome is a better predictor for psoriasis than the metabolic syndrome via an explainable machine learning method — the NHANES survey during 2005–2006 and 2009–2014
Source: Front Endocrinol (Lausanne). 2024 Jun 26;15:1379130. doi: 10.3389/fendo.2024.1379130 (PMC11233539; doi:10.3389/fendo.2024.1379130)
Supplement: Supplementary file 1 [file Image_1.pdf]

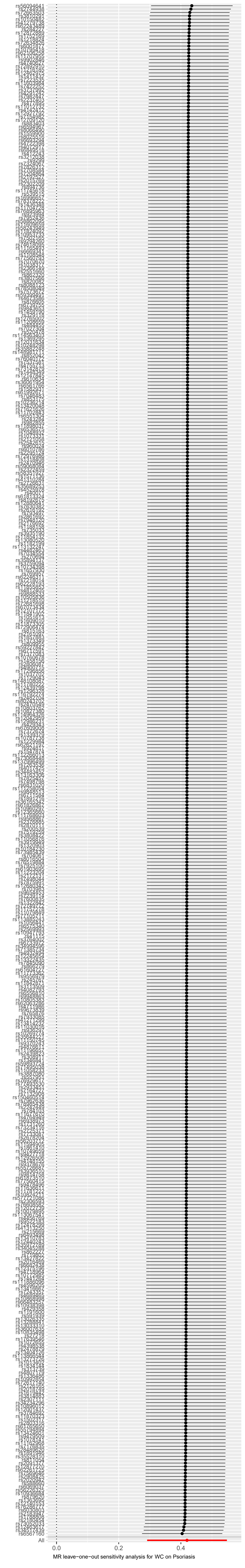
All
0.0
0.2
0.4
MR leave−one−out sensitivity analysis for WC on Psoriasis

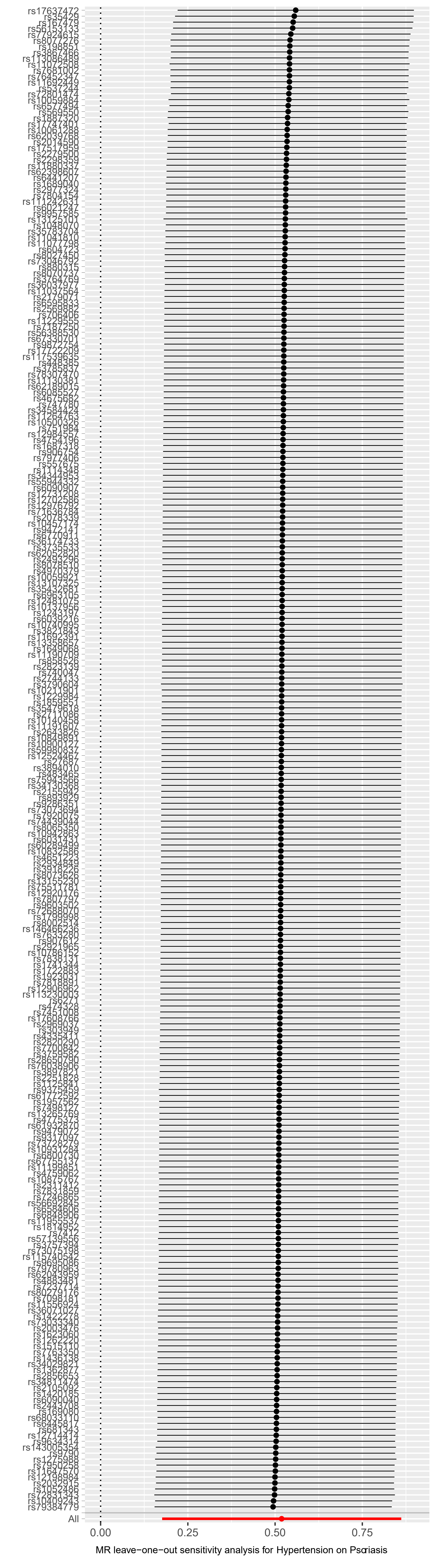
All
0.00
0.25
0.50
0.75
MR leave−one−out sensitivity analysis for Hypertension on Psoriasis

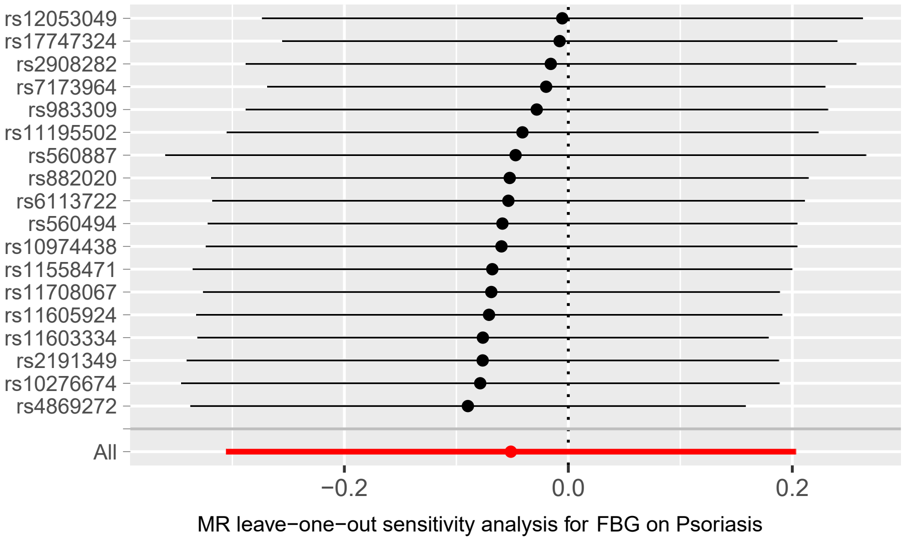

rs12053049
rs17747324
rs2908282
rs7173964
rs983309
rs11195502
rs560887
rs882020
rs6113722
rs560494
rs10974438
rs11558471
rs11708067
rs11605924
rs11603334
rs2191349
rs10276674
rs4869272
All
−0.2
0.0
0.2
MR leave−one−out sensitivity analysis for FBG on Psoriasis

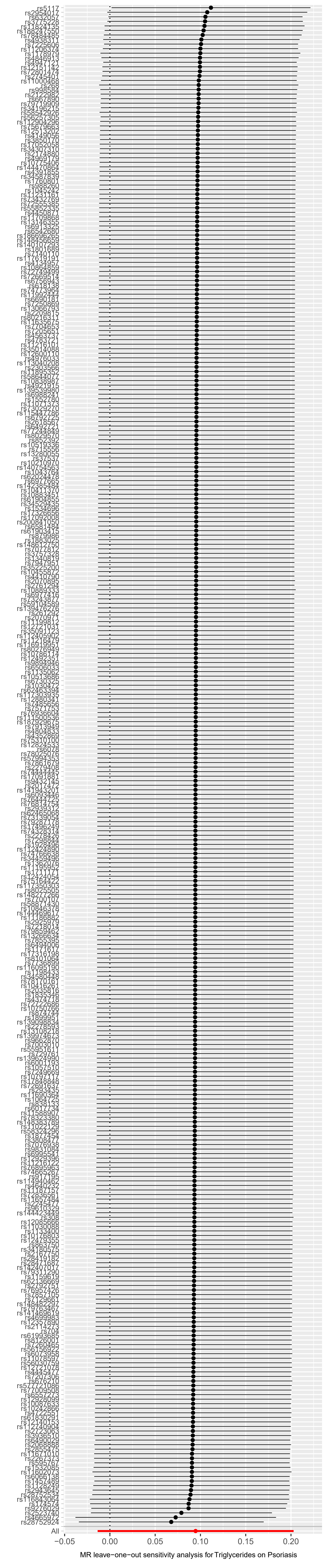
All
−0.05
0.00
0.05
0.10
0.15
0.20
MR leave−one−out sensitivity analysis for Triglycerides on Psoriasis

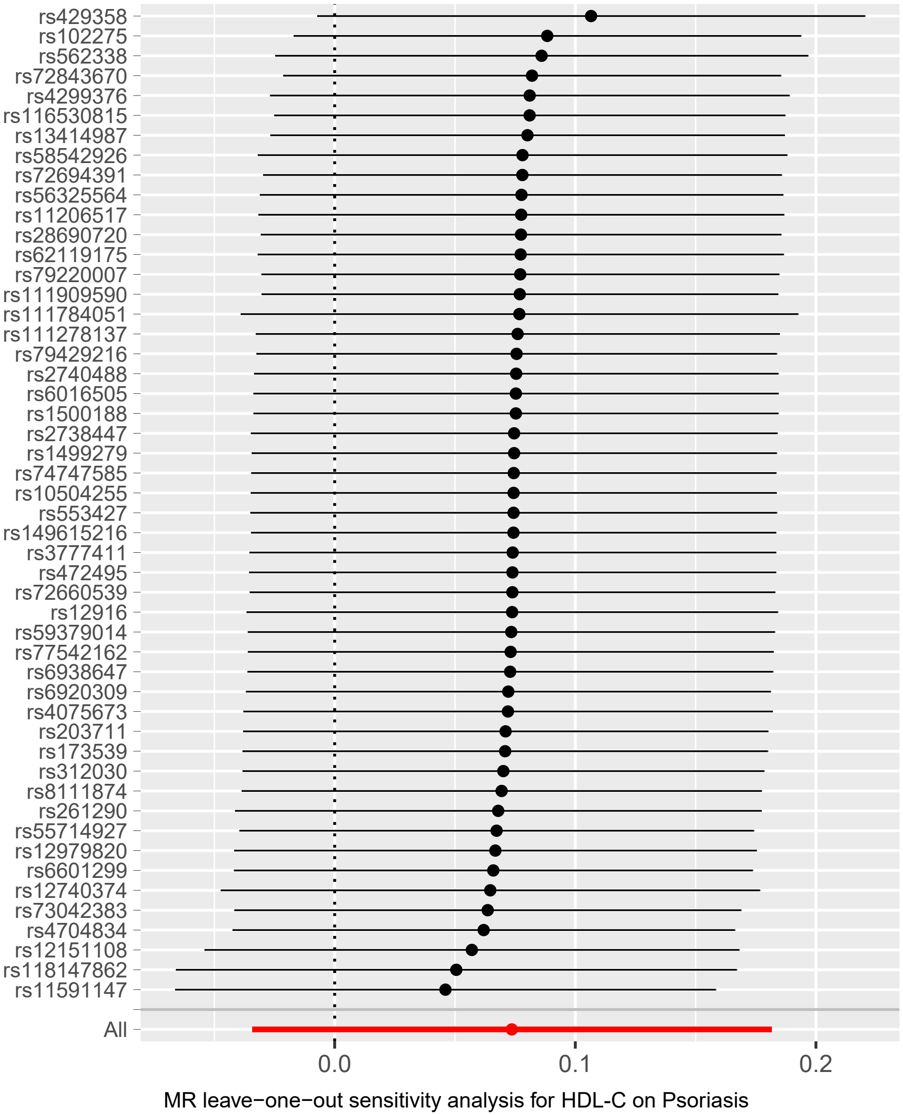
rs429358
rs102275
rs562338
rs72843670
rs4299376
rs116530815
rs13414987
rs58542926
rs72694391
rs56325564
rs11206517
rs28690720
rs62119175
rs79220007
rs111909590
rs111784051
rs111278137
rs79429216
rs2740488
rs6016505
rs1500188
rs2738447
rs1499279
rs74747585
rs10504255
rs553427
rs149615216
rs3777411
rs472495
rs72660539
rs12916
rs59379014
rs77542162
rs6938647
rs6920309
rs4075673
rs203711
rs173539
rs312030
rs8111874
rs261290
rs55714927
rs12979820
rs6601299
rs12740374
rs73042383
rs4704834
rs12151108
rs118147862
rs11591147
All
0.0
0.1
0.2
MR leave−one−out sensitivity analysis for HDL-C on Psoriasis

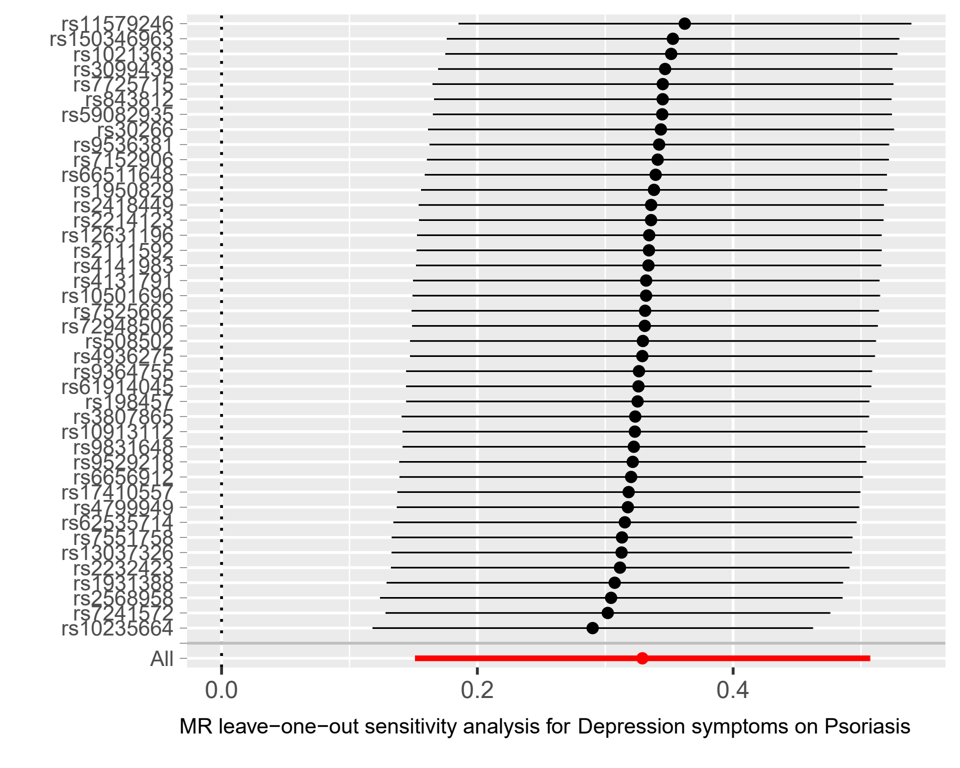

rs11579246
rs150346963
rs1021363
rs3099439
rs7725715
rs843812
rs59082935
rs30266
rs9536381
rs7152906
rs66511648
rs1950829
rs2418449
rs2214123
rs12631196
rs2111592
rs4141983
rs4131791
rs10501696
rs7525662
rs72948506
rs508502
rs4936275
rs9364755
rs61914045
rs198457
rs3807865
rs10913112
rs9831648
rs9529218
rs6656912
rs17410557
rs4799949
rs62535714
rs7551758
rs13037326
rs2232423
rs1931388
rs2568958
rs7241572
rs10235664
All
0.0
0.2
0.4
MR leave−one−out sensitivity analysis for Depression symptoms on Psoriasis

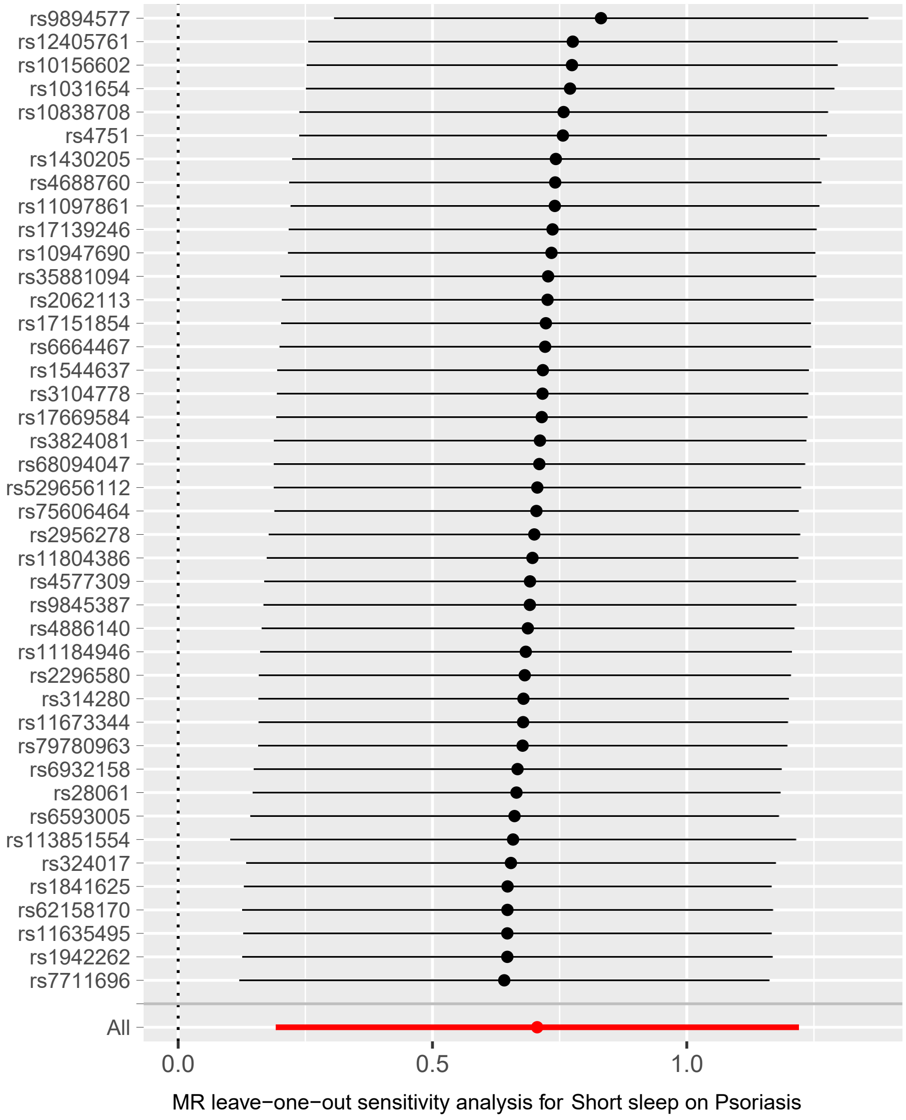
rs9894577
rs12405761
rs10156602
rs1031654
rs10838708
rs4751
rs1430205
rs4688760
rs11097861
rs17139246
rs10947690
rs35881094
rs2062113
rs17151854
rs6664467
rs1544637
rs3104778
rs17669584
rs3824081
rs68094047
rs529656112
rs75606464
rs2956278
rs11804386
rs4577309
rs9845387
rs4886140
rs11184946
rs2296580
rs314280
rs11673344
rs79780963
rs6932158
rs28061
rs6593005
rs113851554
rs324017
rs1841625
rs62158170
rs11635495
rs1942262
rs7711696
All
0.0
0.5
1.0
MR leave−one−out sensitivity analysis for Short sleep on Psoriasis
